# Time: a Constructal viewpoint & its consequences

**DOI:** 10.1038/s41598-019-46980-5

**Published:** 2019-07-18

**Authors:** Umberto Lucia, Giulia Grisolia

**Affiliations:** Dipartimento Energia “Galileo Ferraris”, Politecnico di Torino, Corso Duca degli Abruzzi 24, 10129 Torino, Italy

**Keywords:** Single photons and quantum effects, Statistical physics, thermodynamics and nonlinear dynamics

## Abstract

In the environment, there exists a continuous interaction between electromagnetic radiation and matter. So, atoms continuously interact with the photons of the environmental electromagnetic fields. This electromagnetic interaction is the consequence of the continuous and universal thermal non-equilibrium, that introduces an element of randomness to atomic and molecular motion. Consequently, a decreasing of path probability required for microscopic reversibility of evolution occurs. Recently, an energy footprint has been theoretically proven in the atomic electron-photon interaction, related to the well known spectroscopic phase shift effect, and the results on the irreversibility of the electromagnetic interaction with atoms and molecules, experimentally obtained in the late sixties. Here, we want to show how this quantum footprint is the “origin of time”. Last, the result obtained represents also a response to the question introduced by Einstein on the analysis of the interaction between radiation and molecules when thermal radiation is considered; he highlighted that in general one restricts oneself to a discussion of the *energy* exchange, without taking the *momentum* exchange into account. Our result has been obtained just introducing the momentum into the quantum analysis.

## Introduction

In the last decades, Rovelli^1–3^ introduced new considerations on time in physical sciences. In his *Philosophiae Naturalis Principia Mathematica*^4^, Newton introduced two definitions of time:

- Time is the quantity one introduce when he needs to locate events;
- Time is the quantity which flows uniformly even in absence of events, and it presents a proper topological structure, with a well defined metric.

Then, Special Relativity introduced Newtonian space and time into Minkowski space-time, while, in General Relativity, time definition evolves towards an aspect of the gravitational field^1–3^.

Recently, Loop Quantum Gravity introduced an approach related to General Relativity: there is no preferred clock time, but many clock times measured by different clocks; moreover, clock times undergo standard quantum fluctuations like any other dynamical variable^1–3^.

Moreover, some thermodynamic considerations have been introduced on the measurable clock time in relation to the perception of the human mind^5^. Indeed, the *mind time* is a sequence of images, the results of inflowing stimuli^6^ perceived by sensory organs. It was pointed out how the rate at which changes in mental images are perceived decreases with age, because of several physical features that change with age^7^. This result was obtained by introducing the constructal law of evolution of flow architecture^5,8^.

As a consequence of these approaches on time, a question arises spontaneously: which is the physical nature of time?

In this paper, we will develop only the physical response to this question, by focusing our analysis only on the *physical time* of our Universe.

Recently, we have introduced an analytical approach^9–11^ to irreversibility by considering the continuous interaction between the environmental electromagnetic waves and the matter, due to the continuous non-equilibrium state of reality. In particular we have analysed the absorption-emission of a photon by an atomic electron pointing out the irreversibility presents in this interaction. To do so, we have introduced the Constructal law approach in quantum mechanics.

Constructal law is the thermodynamic theory introduced to optimize the performance of thermo-fluid flow systems by generating geometry and flow structures. So, it has been developed towards a more general thermodynamic theory based on the analysis of the fluxes through the border of the open systems. Consequently, it allows us to understand how natural self-organization and self-optimization evolve^12–22^. This theory introduced a new viewpoint on corals, birds, atmospheric flow, and at machines in general.

Recently, we have introduced just Constructal law to analyse the foundation of atomic physics in relation to quantum irreversibility, in order to obtain a response to the observation of Albert Einstein on the restriction of the energy analysis in relation to the photon-atomic electron interaction without any consideration on the momentum effect^23^. To do so, we have followed the Einstein’s, Schrdinger’s and Gibbs’ considerations on the interaction between photons and atomic electrons, from a microscopic point of view, and matter and thermal radiation from a macroscopic point of view. So, we have highlighted the thermodynamic nature of atoms as open systems with energy levels described by the principal quantum number *n* and in continuous interaction with the environmental electromagnetic radiation, due to non-equilibrium.

Consequently, following the Einstein’s approach, we have considered the electromagnetic wave as a flux of photons, which incomes into the atoms and molecules (our open systems). From a macroscopic point of view, the electromagnetic waves follow the Fermat’s least time principle, while from a microscopic point of view, at atomic level, Bohr described the absorption of a photon by an electron as a resonant process, at a maximum rate of absorption energy. Constructal law allows us to link these approaches together^24–26^, obtaining a completely new point of view for the analysis of the open quantum systems, allowing us to introduce the Gouy-Stodola theorem also to quantum systems^27–29^ with some related applications to complex systems^30–37^.

Our results pointed out that the interaction between a photon and an atomic electron affects the energy level both of the electron and of the center of mass of the atom, and that the variation in momentum and kinetic energy of the center of mass represents the footprint of the interaction.

The aim of this paper is to show that this quantum footprint is the “origin of time”, and in particular that there exists a strict relation between time and entropy generation in the Universe, due to the continuous interaction between electromagnetic radiation (photons) and matter (atomic electrons).

## Results

In any science, but also in everyday life, time represents one of the fundamental quantities; better, there is nothing more fundamental^38^ than time because all the processes in Nature, and all the relations in our life, are defined just in terms of time. Moreover, the notion of time was highlighted to have to emerge through an intimate relationship related to flows^38^. The fundamental question is: which flow must we consider?

Here, we consider that our Universe is always in a disequilibrium state, so, continuous fluxes of energy occur in form of electromagnetic waves (fluxes of photons from a microscopic point of view). Consequently, the analysis of the interactions between these waves and the matter results fundamental in the definition both of irreversibility and of time itself. The thermodynamic theory which focus its research topic on fluxes is the Constructal law. So, in this paper, we have introduced just the Constructal law to analyse the interactions between photons and atomic electrons, relating them to irrevesibility and the consequent definition of time.

Indeed, we have proven that the photon-atomic electron interaction generates an energy footprint, expressed in relation (15), in accordance with the experimental results obtained by Doyle^39^. Moreover, this result represents also a response to the question introduced by Einstein^23^ on the analysis of the interaction between radiation and molecules when thermal radiation is considered; indeed, Einstein highlighted that “in general one restricts oneself to a discussion of the *energy* exchange, without taking the *momentum* exchange into account. One feels easily justified in this, because the smallness of the impulses transmitted by the radiation field implies that these can almost always be neglected in practice, when compared with other effects causing the motion. For a *theoretical* discussion, however, such small effects should be considered on a completely equal footing with more conspicuous effect of a radiative *energy* transfer, since energy and momentum are linked in the closest possible way^23^”.

In the analysis of this quantum process, time never appears. But, it can be defined by introducing the relation between the energy footprint and the power which generates the process, the power of the electromagnetic wave, related to the inflow of photons.

In summary, time results as the footprint of the irreversibility. But irreversibility is described by using entropy. Consequently, we have obtained a thermophysical expression of time by means of the entropy generation, Eq. (20).

## Discussion

The definition and understanding of the nature of time is difficult, but it is so important due to its fundamental role in physics and science. Indeed, all evolves in time.

In Newtonian physics, time is a mathematical variable; any variation of physical quantities refers to it. But, it isn’t something real: it remains only a mathematical variable^40^. Simultaneity and durations of phenomena are absolute: a duration is an abstract property of the whole. So, the life of a radioactive particle is a time interval between two mathematical instants, measured by a universal clock^40^.

Einstein introduced a completely new approach to time in Relativity. In Relativity, the concept of time is a consequence of the postulate of invariance of the speed of light, from which follows the relativity of simultaneity^40^. So, two inertial observers in different states of motion don’t agree on the instants which delimit a time interval of the same event. Instants and durations are dependent on the observer. The physical universe is space-time, a mathematical space continuously filled with ideal clocks^40^. These clocks are all synchronized with respect to a given observer, that measures the duration of a phenomenon through two clocks located in the places where the phenomenon starts and ends. So, another inertial observer, in motion with respect to the first one, doesn’t agree on the space-time coordinates of the same events; consequently, the phenomenon presents a different duration with respect to them. So, the duration of the mean lifetime of a radioactive substance isn’t absolute^40,41^. In relation to the experimental tests on relativistic time dilatation has been pointed out the need of a clear distinction between time dilation effect and clock effect. As a consequence of this distinction, the requirement of the definition of physical time emerges^40^.

In summary, in Newtonian physics, time flows at a constant rate, independently by the observers. On the contrary, in Special Relativity, however, it flows at different rates for different observers. Last, in General Relativity the effect of the time flow is analysed in the presence of gravitational fields. So, in Relativity, time is intimately related with space, emerging the notion of space-time, in which both space and time cannot be considered as separate entities^38^.

As time is incorporated into the proper structure of the space-time, it becomes a geometrical quantity which affects all the natural phenomena, quantum processes included.

In agreement with a Bell^42^ and Pauli^43^, Brown^44^ has highlighted that clocks don’t measure time, but their behaviour correlates with some aspects of spacetime. The relativistic clocks record different durations between two events only if they are in different gravitational potentials^40^.

Moreover, physical systems evolve in the phase space following the path which increases entropy. Related to this increase, the arrow of time emerges, so this represents a topic which links thermodynamic irreversibility with quantum physics.

These considerations highlight the need of a physical definition of time. Our result responds to this requirement. Indeed, the results here obtained in Method Section allow us to point out that time is a footprint of irreversibility due to interaction between the system (the atom in our analysis) and it environment. Our results support a statistical distribution of footprints in accordance with thermodynamics, just based on the macroscopic description of the statistical distribution of physical quantities. So, macroscopic time is the macroscopic description of the statistical distribution of the energy footprints due to the continuous irreversible interactions between electromagnetic radiation and matter (photons and atomic or molecular electrons) due to the non equilibrium state of our Universe.

In particular, the presence of fluxes of energy (and matter) is the way of interaction. So, in our analysis we can consider some cases:

- The atom without interaction can be considered an isolated system and any process inside is completely reversible;
- The atom in interaction with a photon is an open system where fluxes occurs: in this case a photon can be absorbed and the atomic electron can have an energy transition, then the electron can jump down in its fundamental energy state, with a related photon emission: the system is subjected to inflow and outflow of photons;
- The atom in interaction is subjected to the irreversible process of the perturbation of its center of mass: this open atom is irreversible, just because it is in interaction with the environment and it is subjected to fluxes.

Last, from a thermodynamic point of view only duration has sense in relation to the physical time. So, only duration can be measured. Indeed, our definition of time is related to the entropy generation, which is the entropy variation due to irreversibility generated during the interaction.

From these considerations, we can highlight that if the system is subjected to reversible processes, the irreversibility doesn’t occur and in relations (17) and (20) the irreversibility footprint disappears and time becomes null. So, in completely reversible processes the systems move only in the space component of the space time, without having any movement in the time components. So, any completely reversible system seems to be able also to disappear in a space position and to appear in any other space position without spending time.

We wish to suggest that this consideration could be introduced in the analysis of both the tunnel effect and the EPR paradox, which are two very interesting physical topics, but outside of the aim of this paper.

## Methods

At atomic level, the photons can be absorbed by the atomic or molecular electrons, and an electronic energy transition occurs between energy levels of two atomic stationary states. Then, the photons can be also emitted by the excited electrons when they jump down into the energy level of the original stationary state. During this phenomenon, the electrons seem to follow a reversible energetic path, because they come back to the original stationary state of lower energy level. But, as a consequence of the interaction between the atomic or molecule electron and the photon, a footprint occurs in the atom or molecule. The results obtained in refs^9–11^ point out that the interaction between a photon and an electron in an atom affects the energy level both of the electron and of the center of mass of the atom. So, it was analytically proved that the macroscopic irreversibility is no more than a consequence of the microscopic irreversibility due to the interaction photon-electron, or from a macroscopic point of view between the electromagnetic waves and the matter.

### The constructal analysis

At first sight the relation between the energy level gap *E*′ − *E* [J] and the incoming photon energy (*hν* with *h* Planck constant [6.626 × 10^−34^ J s] and *ν* [s^−1^] frequency of the electromagnetic wave) *E*′ − *E* = *hν* is correct insofar as energy conservation is considered. However, a closer examination indicates that it requires a slight modification. But, a photon carriers also a momentum *p*_*photon*_ = *hν*/*c* [kg ms^−1^], with *c* = 299792458 [ms^−1^] velocity of light in vacuum, in addition to the energy *hν* [J], and that both momentum and energy must be conserved in radiative transitions from one state to another. Let us first consider emission by an atom at rest. Initially, before the transition, its momentum is zero. After the transition the atom must recoil with a momentum equal and opposite to the momentum of the photon; that is, 0 = **p**_*atom*_ + **p**_*photon*_ or, in magnitude,

1 $${p}_{atom}={p}_{photon}=h\nu /c$$

Now, let us consider the energy conservation. Initially we have an atom at rest in a stationary state of energy *E*_*i*_ [J], and after the transition an atom in a stationary state of energy *E*_*f*_ [J] with kinetic energy ${p}_{atom}^{2}/2M$ [J] and a photon of energy *hν* [J]. Therefore energy conservation requires that^45^

2 $${E}_{i}={E}_{f}+\frac{{p}_{atom}^{2}}{2M}+h\nu $$

or,

3 $${E}_{i}-{E}_{f}=h\nu (1+\frac{h\nu }{2M{c}^{2}})$$

In general, *hν* [J] is smaller than *Mc*^2^ [J], so:

4 $$h\nu =({E}_{i}-{E}_{f}){(1+\frac{h\nu }{2M{c}^{2}})}^{-1}=({E}_{i}-{E}_{f})(1-\frac{h\nu }{2M{c}^{2}}),$$

In the last term we can replace *hν* by *E*_*i*_ − *E*_*f*_ resulting in

5 $$h\nu ={E}_{i}-{E}_{f}-\frac{{({E}_{i}-{E}_{f})}^{2}}{2M{c}^{2}}$$

where the last term is essentially the recoil energy of atom. Therefore, in the emission process, the energy emitted by photon is slightly less than the difference between the two energy levels of the emitter (atom, molecule, or nucleus). The difference is the recoil energy of the emitter.

On the other hand, for the absorption process we can follow a similar approach, obtaining^45^

6 $${E}_{i}+h\nu ={E}_{f}+\frac{{p}_{atom}^{2}}{2M}$$

since there is now a photon in the initial state but not in the final state. Conservation of momentum requires that **p**_*atom*_ = **p**_*photon*_. So, when we use the same approximation as above, it follows

7 $$h\nu =({E}_{f}-{E}_{i}){(1-\frac{h\nu }{2M{c}^{2}})}^{-1}=({E}_{f}-{E}_{i})(1+\frac{h\nu }{2M{c}^{2}})$$

or

8 $$h\nu ={E}_{f}-{E}_{i}-\frac{{({E}_{f}-{E}_{i})}^{2}}{2M{c}^{2}}$$

Therefore, for absorption to take place, the energy of the absorbed photon must be slightly greater than the energy difference between the two levels of the absorber to account for the kinetic energy of the recoiling absorber.

### Time as consequence of constructal considerations

The fundamental state function, before the interaction, solution of the Schrdinger equation can be obtained by the quantum mechanics^46,47^:

9 $$\psi ({\bf{r}},{\bf{R}})=\varphi ({\bf{r}})\,\vartheta ({\bf{R}})$$

where *φ* is the wave function of the electron, **r** = **r**_*N*_ − **r**_*e*_ [m] are the relative coordinates, with **r**_*N*_ [m] the coordinates of the atomic nucleus and **r**_*e*_ [m] the coordinates of the atomic electron, and^9–11^:

10 $$\vartheta ({\bf{R}})=\frac{1}{{(2\pi )}^{3/2}}\exp (i{\bf{k}}\cdot {\bf{R}})$$

11 $${\bf{k}}=\sqrt{\frac{2M}{\hslash }{E}_{CM}}{{\bf{u}}}_{CM}$$

where **u**_*CM*_ is the versor of the nucleus momentum, *E*_*CM*_ = **P**^2^/2*M* [J] is the kinetic energy of the center of mass, **R** = (*m*_*N*_**r**_*N*_ + *m*_*e*_**r**_*e*_)/(*m*_*N*_ + *m*_*e*_) [m] is the coordinate of the center of mass before photon-atomic electron interaction, *M* is the total mass of the atom, *m*_*e*_ is the mass of the electron and *m*_*N*_ is the mass of the nucleus, **P** is the momentum of the center of mass, ℏ = *h*/2*π* where *h* is the Planck constant, and *u*_*CM*_ is the versor of the momentum of the nucleus.

Then, the photon incomes to the atomic electron which jumps from the fundamental state into an excited energy state and then it jumps down to the fundamental state, with the emission of a new photon. The fundamental state function, after this interaction, solution of the Schrdinger equation can be obtained by the quantum mechanics^9–11,46,47^:

12 $${\psi }_{f}({\bf{r}},{\bf{R}})=\varphi ({\bf{r}})\,{\vartheta }_{f}({\bf{R}})$$

with *φ* wave function of the electron and^9–11,45^:

13 $${\vartheta }_{f}({\bf{R}})=\frac{1}{{(2\pi )}^{3/2}}\exp (i{\bf{k}}^{\prime} \cdot {\bf{R}})$$

14 $${\bf{k}}^{\prime} =\sqrt{\frac{2M}{\hslash }({E}_{CM}+\frac{{m}_{e}}{M}\,{E}_{ph})}({{\bf{u}}}_{CM}+{{\bf{u}}}_{c})$$

where *E*_*ph*_ is the energy of the incoming photon, and **u**_*c*_ = **c**/*c* is the versor of propagation of the electromagnetic wave, with **c** the velocity of light and *c* its value.

Recently, we have suggested a quantum thermodynamic approach to this interaction photon-atomic electron interaction, proving that this atomic process leaves the footprint^9–11^:

15 $${E}_{ftp}={\rm{\Delta }}{E}_{ph}={\rm{\Delta }}{E}_{CM}=\langle \psi ({\bf{r}},{\bf{R}})|H|{\psi }_{f}({\bf{r}},{\bf{R}})\rangle =\frac{{m}_{e}}{M}\,{E}_{ph}$$

where *H* is the Hamiltonian of the photon-atomic electron interaction, i.e. from a macroscopic point of view, the interaction between electromagnetic wave and matter.

But, a quantity doesn’t appear in this quantum approach of the transition between atomic steady states: it is the time. But, something happens that requires to introduce it; indeed, this process is completely irreversible, because this phase shift cannot be inverted, as it is well known in spectroscopy^39,47–49^. So, we must introduce a quantity which shows this irreversibility. To do so, we consider that a photon is the quantum of the electromagnetic wave, so we can obtain its power by considering the power of the electromagnetic wave^50^:

16 $$\dot{Q}=(\frac{1}{2}{\varepsilon }_{0}c{E}_{el}^{2}+\frac{1}{2{\mu }_{0}}c{B}_{m}^{2})A$$

where *E*_*el*_ [Vm^−1^] is the electric field, *B*_*m*_ [T] is the magnetic field, *c* is the velocity of light, *ε*_0_ = 8.854 × 10^−12^ [N m^2^A^−2^s^−2^] is the electric permittivity in vacuum and *μ*_0_ = 4*π* × 10^−7^ [Hm^−1^] is magnetic permeability in vacuum, and *A* [m^2^] is the area of the interaction surface considered, i.e. the area of the atomic surface, as usually considered in electromagnetism.

In the interaction between the photon and the bond electron, from a thermodynamic point of view, $\dot{Q}$ [W] is the incoming power into an open system (here the atom). But, always from a thermodynamic point of view, *E*_*ftp*_ [J] in Equation (5) is the energy wasted during a process (here the excitation-de-excitation process of the atomic electron). Consequently, time can be introduced as the quantity which expresses the irreversibility of the interaction between electromagnetic wave and the matter, i.e. the interaction of excitation and de-excitation of the atomic electrons in interaction with the photons, obtaining the analytical definition^51^:

17 $$t=\frac{{E}_{ftp}}{\dot{Q}}=\frac{\frac{{m}_{e}}{M}\,{E}_{ph}}{(\frac{1}{2}{\varepsilon }_{0}c{E}_{el}^{2}+\frac{1}{2{\mu }_{0}}c{B}_{m}^{2})A}$$

Now, we wish to extend this microscopic result to the macroscopic approach. To do so, we follow the same approach used in analytical mechanics, defining a face space Ω in relation to general independent quantities $(q,\dot{q})$. So, we introduce the phase space ${\rm{\Omega }}=\{(S,\dot{S}),S\subseteq {{\mathbb{R}}}^{+}\cup \{0\},\dot{S}\subseteq {{\mathbb{R}}}^{+}\cup \{0\}$, where *S* [JK^−1^] is the entropy and $\dot{S}$ [WK^−1^] is the entropy rate, exchanged by the system, respectively. In this way any system evolves by a path *γ* ∈ Ω.

It is always possible to link entropy and entropy rate to the physical quantities exchanged by a real system by using the first law and the second law of thermodynamics, together with the Theorem of Gouy-Stodola^18^.

Consequently, considering the Constructal law approach the variation of *S* and $\dot{S}$ is related to the fluxes of energy and matter between open systems, so, we can write that^9^:

18 $$\frac{{m}_{e}}{M}{E}_{ph}={T}_{0}S$$

and also^52^:

19 $$\frac{1}{2}{\varepsilon }_{0}c{E}_{el}^{2}+\frac{1}{2{\mu }_{0}}c{B}_{m}^{2}=\frac{{T}_{0}}{A}\dot{S}$$

so, it follows:

20 $$t=\frac{S}{\dot{S}}$$

Consequently, time is the footprint of irreversibility of any subset of the Universe, due to the fluxes of energy and mass among them.

We wish to highlight that the environmental temperature *T*_0_ fundamental in the use of the Gouy-Stodola theorem disappears in the evaluation of time interval. Consequently, it is not necessary its evaluation in this approach. It is an arbitrary quantity. Last, the relation (20) measures a duration of the interaction *τ* = *t* − *t*_0_ [s], where *t* [s] is the instant at the start of the interaction and *t*_0_ [s] is the instant at the end of the interaction. If we consider *t*_0_ = 0 [s] for each interaction then *τ* = *t* [s] is the time.
